# Erratum

**DOI:** 10.1002/ehf2.14267

**Published:** 2022-12-15

**Authors:** Antonia Camporeale

In the paper by Disabato *et al*.,^1^ the funding statement BIBLIOSAN is missing in the published version.

The funding details has been added in the Acknowledgement as follows:
